# Is S100B Involved in Attention-Deficit/Hyperactivity Disorder (ADHD)? Comparisons with Controls and Changes Following a Triple Therapy Containing Methylphenidate, Melatonin and ω-3 PUFAs

**DOI:** 10.3390/nu15030712

**Published:** 2023-01-31

**Authors:** Miriam Ouadih-Moran, Antonio Muñoz-Hoyos, Luis D’Marco, Antonio Molina-Carballo, Isabel Seiquer, Ana Checa-Ros

**Affiliations:** 1 Department of Pediatrics, Torrevieja University Hospital, 03186 Alicante, Spain; 2 Department of Pediatrics, School of Medicine, University of Granada, 18016 Granada, Spain; 3 Department of Medicine & Surgery, Faculty of Health Sciences, Universidad Cardenal Herrera—CEU, CEU Universities, 46115 Valencia, Spain; 4 Department of Physiology and Biochemistry of Animal Nutrition (EEZ-CSIC), Camino del Jueves, 18100 Granada, Spain; 5 Aston Institute of Health and Neurosciences, School of Life & Health Sciences, Aston University, Birmingham B4 7ET, UK

**Keywords:** ADHD, S100B, attention, methylphenidate, melatonin, ω-3 PUFAs

## Abstract

Background: Increasing evidence supports a neuroinflammatory basis in ADHD damaging glial function and thereby altering dopaminergic (DA) neurotransmission. Previous studies focusing on the S100B protein as a marker of glial function have shown contradictory results. We conducted a clinical trial to investigate differences in S100B levels between ADHD patients and controls, as well as observe gradual changes in S100B concentrations after a triple therapy (TT) containing methylphenidate (MPH), melatonin (aMT) and omega-3 fatty acids (ω-3 PUFAs). Methods: 62 medication-naïve children with ADHD (ADHD-G) and 65 healthy controls (C-G) were recruited. Serum S100B was measured at baseline (T0) in ADHD-G/C-G, and three (T3) and six months (T6) after starting TT in the ADHD-G, together with attention scores. Results: A significant increase in S100B was observed in the ADHD-G vs. C-G. In the ADHD-G, significantly higher S100B values were observed for comparisons between T0–T3 and between T0–T6, accompanied by a significant improvement in attention scores for the same timepoint comparisons. No significant differences were found for S100B between T3–T6. Conclusion: Our results agree with the hypothesis of glial damage in ADHD. Further studies on the link between DA and S100B are required to explain the transient increase in S100B following TT.

## 1. Introduction

Attention-deficit and/or hyperactivity disorder (ADHD) is a common neurodevelopmental disorder in children and adolescents, characterized by the impairing presence of inattention, hyperactivity, and impulsivity [1]. Its worldwide prevalence has been reported to be around 5.3% [2], with a probability of around 30–50% to become chronic and persist into adulthood [3]. ADHD is clinically diagnosed when symptoms, appearing before 12 years of age, meet six or more of the inattention or hyperactivity/impulsivity criteria, or both, allowing the distinction of three different ADHD presentations (inattentive, hyperactive, and impulsive, or combined) [4]. To date, no biochemical or neuroimaging features have been confirmed to have diagnostic value for ADHD [5,6]. Frequently comorbid with other psychiatric and neurological disorders (such as sleep disorders and autism spectrum disorders), ADHD may become a substantial burden for not only the patient, but for the family and the community as well [7].

The pathophysiology of ADHD is not completely understood. Core symptoms have been linked to disruptions in the maturation of the cerebellum, caudate and prefrontal cortex (PFC). These areas create a network involved in the regulation of executive functions (attention, working memory, behavioral inhibition, and planning) [8,9,10], and are biochemically mediated by catecholaminergic neurotransmitters, such as dopamine (DA) and norepinephrine (NE) [11,12]. Deficits in executive functions, secondary to abnormalities of dopaminergic pathways, are shown by patients with ADHD [13,14,15]. 

The mechanisms leading to the catecholaminergic disruption observed in this disorder are multifactorial [16] and yet to be clarified. Increased glial activity has been reported in murine models of ADHD [17,18], suggesting involvement of glial cells in the functional and structural changes preceding ADHD symptoms. The glial function is essential for neuronal pruning and firing [6,19], network connectivity during development, and the secretion of pro-inflammatory and anti-inflammatory cytokines [20]. 

The cytokine-related neurotrophin S100B, mainly secreted by astrocytes and oligodendrocytes, is a well-known biomarker of glial function [21]. At nanomolar concentrations, S100B is involved in calcium and glutamate uptake, energy metabolism and neuronal plasticity mediated by serotonin (5-HT) [22], a neurotransmitter that interacts with DA to regulate impulsivity and sensitivity to emotions [23]. However, at micromolar concentrations, S100B acts as a damage-associated molecular pattern (DAMP) that interacts with the receptor for advanced glycation-end products (RAGE), activating multiple signaling pathways in inflammation via the mitogen-activated protein kinase (MAPK) [24,25,26,27]. The link between S100B and catecholaminergic neurotransmitters has been underlined by diverse studies: S100B might modulate the expression of DA D2 receptor [28,29]; in rat models of Parkinson’s disease, depletion of striatal DA was followed by morphological changes in S100B-expressing astrocytes and increased S100B concentrations [30]; S100B-knockout mice were found to have significantly decreased levels of NE in the neocortex and hippocampus in comparison with wild-type mice [31].

Variations in S100B levels have been associated with neuropsychiatric diseases for which a redox imbalance and inflammatory basis are proposed [32]. Significant differences in S100B and high mobility group box 1 (HMGB1) concentrations were observed between patients with schizophrenia and controls [33]; in fact, an inverse correlation was found between S100B levels and the Positive and Negative Syndrome Scale (PANSS) score at discharge after acute relapses of paranoid schizophrenia [34]. In transgenic mice, higher serum S100B levels were related to brain-region-specific and sex-dependent amyloid-β deposition, suggesting a role of S100B in the pathophysiology of Alzheimer’s disease [35,36]. In children with autism spectrum disorders (ASD), a neurodevelopmental condition highly related to ADHD, peripheral blood S100B concentrations were reported to be significantly increased compared with controls [37,38].

Nowadays, increasing evidence supports a neuroinflammatory origin of ADHD, as an association was found with single-nucleotide polymorphisms (SNPs) in genes involved in angiogenesis and inflammation [39]. Moreover, an inflammation-related genomic overlap was observed between ADHD and other psychiatric disorders, such as depression [40]. In a more recent study conducted on women during the third trimester of pregnancy, plasma concentrations of nuclear factor kappa B (NF-kB)-related inflammatory markers were related to the appearance of ADHD symptoms in their offspring during childhood [41]. The first attempt to establish an association between S100B levels and ADHD symptoms was made by Oades et al. [42] on a group of 21 medication-naïve children with ADHD, 21 typically developing controls, 14 ADHD children under medication, and 7 healthy siblings. No significant differences in serum S100B levels were observed between groups, although a trend towards decreased S100B levels was described in patients with internalizing symptoms (e.g., anxiety, depression). In further linear regression analyses, the same authors reported an inverse association between ADHD total symptom ratings and S100B levels [43]. However, a cross-sectional study conducted on school-aged children with heavy metal-related ADHD in China revealed a significantly positive correlation between serum S100B levels and ADHD-like symptoms, although the correlation proved negative for the inattention and hyperactivity/impulsivity indexes in the Conners’ rating scale for teachers [44]. The results obtained by our group in a further clinical trial agreed with Oades et al. [42], as no significant differences in serum S100B levels between ADHD children and controls were found; nevertheless, we also revealed that patients with depressive symptoms were prone to show higher S100B levels [45]. Given the contradictory results obtained by the few studies measuring S100B in patients with ADHD, further exploration of the involvement of this protein in ADHD could help understand the pathophysiology of this disorder.

Psychostimulants, and particularly the different formulations of methylphenidate (MPH), continue to be the first-line pharmacological treatment for ADHD. MPH blocks the presynaptic DA and NE transporters that respectively reuptake these neurotransmitters, thus facilitating catecholaminergic neurotransmission [46]. However, MPH is not exempt from frequently provoking common side-effects, such as insomnia and lack of appetite [47]. Melatonin (aMT) has largely been used as a safe therapeutic option to alleviate sleep-onset insomnia in children with ADHD under MPH treatment [48,49]; additionally, it has proved to be a neurotrophic factor and an anti-inflammatory/antioxidant molecule [50,51]. Omega-3 polyunsaturated fatty acids (ω-3 PUFAs) have also been reported to be beneficial as non-pharmacological adjuvant therapies for ADHD, given their properties in combating inflammation [52,53].

Based on the interaction with DA/NE neurotransmitters and the pro-inflammatory actions of S100B, we support that this protein may act as a mediator in the neuroinflammatory mechanisms leading to the catecholaminergic disruption preceding ADHD symptoms. Accordingly, we hypothesized that: (1) S100B concentrations in pediatric patients with ADHD should differ from controls; and (2) variations in S100B levels should accompany the clinical response of ADHD patients with a triple therapy consisting of MPH, aMT and ω-3 PUFAs. In this dual three-month-phase clinical trial on children and adolescents with ADHD, we aimed at (1) evaluating differences in serum S100B levels between ADHD patients and healthy controls; and (2) assessing gradual changes in both serum S100B concentrations and clinical symptoms (as per attention scores) after initiating treatment with a combination of MPH, aMT and ω-3 PUFAs.

## 2. Materials and Methods

### 2.1. Study Design

We designed a two-year-duration, single-center, open-label clinical trial on children and adolescents with ADHD. 

### 2.2. Population

Medication-naïve children and adolescents between 6 and 15 years old who had recently been diagnosed with ADHD in accordance with the DSM-5 criteria [4] and were considered candidates to receive treatment with psychostimulants [54] were invited to participate in the study. They were recruited from the Neuropediatrics Unit at San Cecilio University Hospital (Granada, Spain). Informed consent and assent were respectively gathered from parents/guardians and patients before inclusion. Exclusion criteria were: (1) current treatment with psychostimulants, melatonin, or ω-3 PUFAs; (2) patients suffering from heart disease; (3) patients diagnosed with glaucoma; (4) presence of neuropsychiatric, metabolic, or endocrine disorders able to justify the current symptoms; (5) refusal to participate. 

A control group composed of age- and sex-matched healthy children was also recruited following the same exclusion criteria. They were children and adolescents attending the hospital for routine blood extraction and free of cardiologic, neuropsychiatric, metabolic, or endocrine disorders.

### 2.3. Methods

#### 2.3.1. Clinical Methods

Initial clinical interview and physical examination were supported by the application of the following questionnaires as part of the diagnostic workup for ADHD: (1) the NICHQ Vanderbilt Parent and Teacher Assessment Scales [55,56]; (2) the Children’s Depression Inventory (CDI) [57] in children >7 years old; (3) the Wechsler Intelligence Scale for Children, 4th edition (WISC-IV) [58]; and (4) the Magallanes Scale of Visual Attention (MSVA) [59].

The CDI is a self-report assessment of depression for children and adolescents between 7–17 years old. It contains two subscales: negative mood and negative self-esteem. For each item, the respondent is presented with three choices that correspond to three levels of symptomatology: 0-absence of symptoms; 1-mild or probable symptom; 2-definite symptoms. A quantitative cut-off > 18 (normative T-score > 50) is considered pathological [57]. The CDI allowed us to detect the presence of depressive (internalizing) symptoms. 

MSVA is a psychometric tool designed to evaluate several attentional functions: focusing, maintaining, coding and stability. The patient is exposed to visual stimuli during a certain period (6 min in children under 9 years of age and 12 min in those older) while a simple motor task is performed by asking them to identify the figure of a man described within a grid full of distracting stimuli in the form of human figures in other postures or rotations. By quantifying the number of correct responses, commission errors, and omission errors, two variables are obtained: sustained visual attention (SA) [SA: (correct responses + omissions)/total n° of correct responses)] and quality of attention [QA: (correct responses – omissions − commission errors)/(correct responses + omissions)] [59]. While the SA represents the capacity to focalize and code visual stimuli during this time, the QA refers to the efficacy in focalizing and coding visual stimuli [60]. Omission errors are derived from inattention, whereas commission errors are associated with impulsivity and hyperactivity [61]. The MSVA-associated computer program (TIPI-SOFT) provides the respective percentile scores of SA and QA. MSVA is currently considered a valid tool to aid in the diagnosis of ADHD [62].

#### 2.3.2. Analytical Methods 

Fasting venous blood samples were collected in the morning (08.00–09.00 h). After centrifugation, the serum was separated and frozen at −80 °C. Protein S100B was measured through the sandwich enzyme-linked immunosorbent assay (Sandwich ELISA), which uses a capture antibody and a detection antibody conjugated with isoluminol. The equipment was provided by LIAISON^®^ (Diasorin S.p.A., Saluggia, Vercelli, Italy). The assay sensitivity was 30 µg/L.

#### 2.3.3. Therapeutic Intervention

The following combination (triple therapy) was administered: (1) prolonged-release MPH (OROS formulation), given in the morning at an initial dose of 0.7 mg/kg/day that was adjusted according to response and treatment; (2) aMT, administered at 3 mg/night 30 min before usual bedtime; and (3) a combination of ω-3 PUFAs, consisting of eicosapentaenoic acid (EPA) at 70 mg/day and docosahexaenoic acid (DHA) at 250 mg/day. 

### 2.4. Patient Follow-Up 

**Figure 1:**
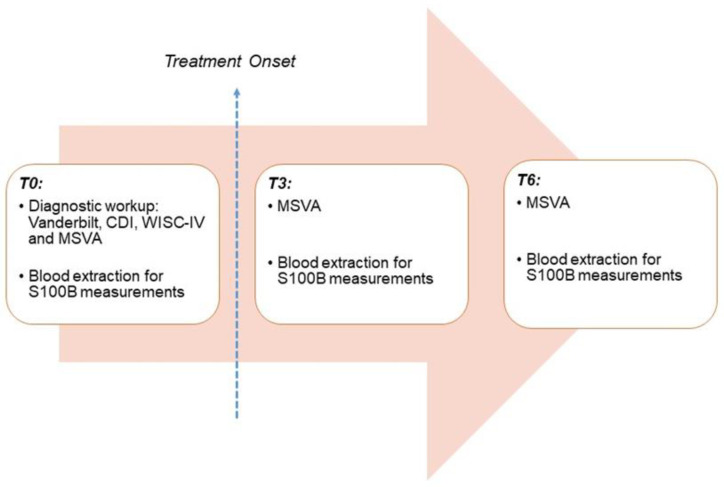
Schematic representation of study protocol. CDI: Children’s Depression Inventory; WISC-IV: Wechsler Intelligence Scale for Children, 4th edition; MSVA: Magallanes Scale of Visual Attention.

We decided to use the QA/SA scores from the MSVA as objective indicators of the clinical response to therapy, based on previous studies reporting on the effects of MPH and ω-3 PUFAs on omission and commission errors [53,61]. Application of MSVA and blood extractions for S100B measurement were performed at three specific time points over a six-month follow-up period: at baseline (T0); three months after starting treatment (T3); and six months after treatment onset (T6) (Figure 1). The timeframe was chosen based on the accretion time of ω-3 PUFAs, which is reported to be higher than eight weeks [63]. 

### 2.5. Statistical Methods

Descriptive data were presented as mean (standard deviation, SD). Comparisons were made for S100B levels between the ADHD group and the control group, as well as for the values of SA/QA and S100B in ADHD patients at T0–T3–T6. For comparative analyses, the Kolmogorov-Smirnov test was applied to verify the normality assumption. Parametric tests were used for comparisons in S100B levels: a Student’s *t*-test to make comparisons between controls and ADHD patients, and an analysis of variance (ANOVA) and Bonferroni post-hoc test for comparisons across time within the ADHD group. The non-parametric Friedman test was applied to compare SA/QA scores at T0–T3–T6 in the ADHD group. The significance level was set at α = 0.05. 

The Statgraphics Centurion version XVII software (Statpoint Technologies, Inc, Warrengton, VA, USA) was used for statistical calculations.

### 2.6. Ethical Considerations

The study protocol was approved by the Ethics Committee of Biomedical Research at San Cecilio University Hospital (Granada, Spain). All procedures were carried out in accordance with the Declaration of Helsinki, as revised in 2013 [64].

## 3. Results

### 3.1. Population

Sixty-two children and adolescents with ADHD participated in the study. The ADHD group was composed of 41 males (66.13%) and 21 females (33.87%), with a mean age of 9.26 (2.11) years. In accordance with the DSM-5 criteria [4], 32 patients (51.61%) were classified as an inattentive presentation (ADHD-I) and 30 (48.38%) as a combined presentation (ADHD-C). Three patients (4.84%) obtained a score > 18 (T-score > 50) in the CDI. Regarding comorbidities, ten ADHD patients (16.13%) were diagnosed with dyslexia, four patients (6.45%) with primary enuresis, two children (3.22%) with epilepsy, one patient (1.61%) with migraine, while another (1.61%) suffered from tics. 

The control group was composed of 65 patients, 42 males (64.61%) and 23 females (35.38%), with a mean age of 9.34 (2.30) years. Table 1 summarizes the demographic and clinical information of participants.

### 3.2. MSVA Values

**Figure 2:**
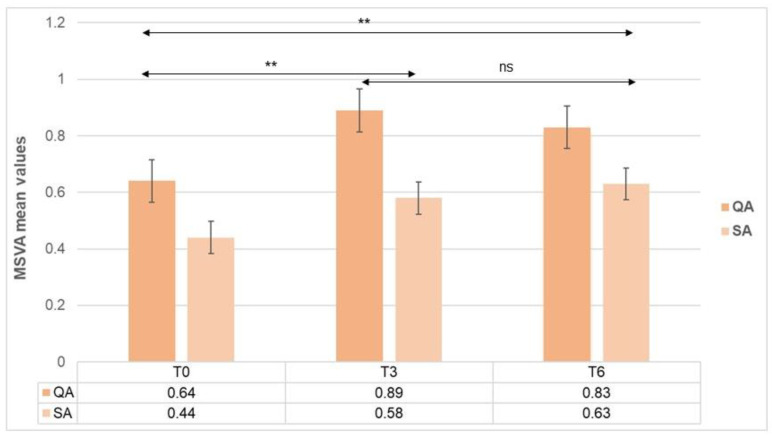
Comparative analysis in the values of quality of attention (QA) and sustained attention (SA) between the different time points in the ADHD group. MSVA: Magallanes Scale of Visual Attention; ns: non-significant; ** *p* < 0.001.

A significant increase was observed for both the QA and SA when comparing their basal values at T0 with those obtained at T3 [QA mean score: 0.64 (0.24) at T0 vs. 0.89 (0.12) at T3 (*p* < 0.001); SA mean score: 0.44 (0.14) at T0 vs. 0.58 (0.16) at T3 (*p* < 0.001)] and at T6 [QA mean score: 0.64 (0.24) at T0 vs. 0.83 (0.33) at T6 (*p* < 0.001); SA mean score: 0.44 (0.14) at T0 vs. 0.63 (0.17) at T6 (*p* < 0.001)]. No significant differences were observed, however, for comparisons of QA and SA values between T3 and T6 (Figure 2).

The improvement observed in QA/SA values as given by the MSVA was supported by the improvement of symptoms and academic performance referred by parents/guardians and teachers.

### 3.3. Serum S100B Concentrations

**Figure 3:**
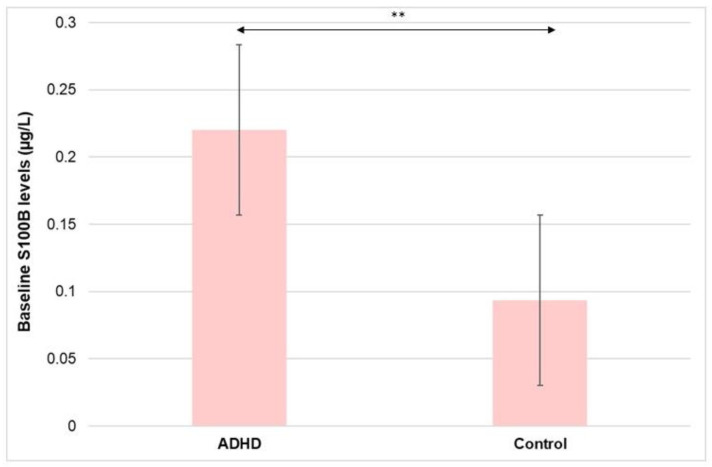
Comparative analysis of serum S100B levels between ADHD patients and the control group at baseline (T0). ** *p* < 0.001.

Serum S100B levels were significantly reduced in the control group compared to ADHD patients at T0 [control group: 0.0938 (0.035) μg/L vs. ADHD group: 0.2204 (0.089) μg/L at T0 (*p* < 0.001)] (Figure 3).

**Figure 4:**
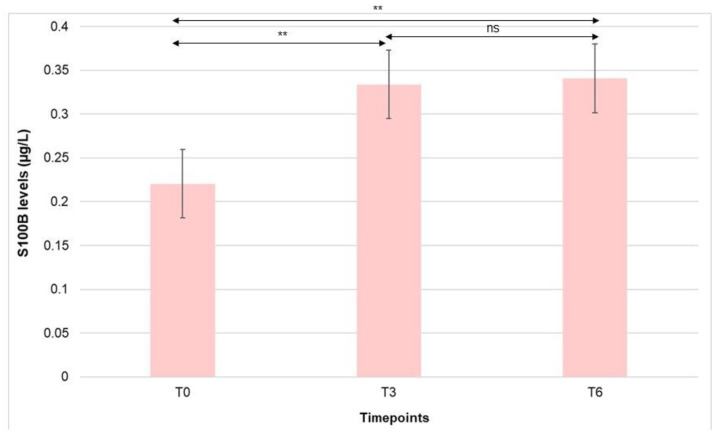
Comparative analysis of serum S100B levels at the different time points (T0, T3, T6) in the ADHD group. ns: non-significant; ** *p* < 0.001.

When comparing serum S100B levels at different time points in the ADHD group, a significant increase was observed at T3 in relation to T0 [S100B mean values: 0.2204 (0.089) μg/L at T0 vs. 0.3339 (0.1447) at T3 (*p* < 0.001)], as well as significantly higher S100B concentrations at T6 compared to T0 [S100B mean values: 0.2204 (0.089) μg/L at T0 vs. 0.3408 (0.1527) μg/L at T6 (*p* < 0.001)]. No significant differences in S100B values were found between T3 and T6 (Figure 4).

We additionally assessed differences in peripheral S100B levels between the patients classified into different ADHD presentations (ADHD-I and ADHD-C). Intergroup comparisons (ADHD-I vs. ADHD-C) across time revealed no significant differences in S100B levels. However, when analyzing the evolution of S100B values across time for the patients included in the same ADHD presentation (intragroup analysis), differences between T0–T3, T0–T6, and T3–T6 followed a similar pattern to the one observed for the whole group (Table 2).

## 4. Discussion

In this clinical trial on a sample of medication-naïve children and adolescents with ADHD, peripheral S100B levels were measured with two main objectives: (1) to establish comparisons with healthy children as controls; and (2) to observe gradual differences in serum S100B values in the ADHD group before (T0) and after (T3–T6) treatment with a triple therapy containing MPH, aMT and ω-3 PUFAs (EPA/DHA). Secondarily, we also reported the clinical effects obtained with this therapy based on the attention measures obtained in the MSVA and the reports from parents/guardians and teachers. At baseline (T0), we found significantly higher levels of S100B protein in the ADHD group compared to control participants. Focusing on the patients with ADHD, a significant increase in S100B values was observed when comparing the levels of this protein before treatment onset with those three and six months after starting therapy. However, S100B values at T3 and T6 were similar. The two components of attention, QA and SA scores, also showed a significant improvement after three and six months of therapy in relation to baseline values (T3 vs. T0 and T6 vs. T0), with no significant changes observed when comparing QA/SA scores between three and six months after treatment onset. 

When comparing S100B values between ADHD patients and controls, our results differed from the first studies presented by Oades et al. [42] and later by our group [45], in which no significant differences were observed between participants with ADHD and healthy children; indeed, an inverse relationship was reported between peripheral S100B levels and ADHD symptoms [43]. According to this, Oades et al. [42] supported that the glial damage occurring in ADHD should be differentiated from the one taking place in other neuroinflammatory-based processes, such as schizophrenia [33,34,65], depression [66] or Alzheimer’s disease [35,36]. Instead, astrocytic alteration in ADHD would be characterized by decreased release of S100B, reflecting insufficient energy supply to neurons [67]. Certainly, in the investigations conducted by Oades et al. [42] and Fernández-López et al. [45], the control group was partly or totally composed of healthy siblings to the ADHD participants; therefore, genetic similarities in S100B values should be taken into consideration. Additionally, these authors excluded patients with neurologic comorbidities, including epilepsy. In our study, the ADHD sample included patients with diverse comorbidities, such as dyslexia and epilepsy. Certainly, this factor could have influenced our results and the discrepancies found with the previous studies. Associations between S100B and candidate genes for dyslexia have been reported [68,69]; in patients suffering from status epilepticus, significantly-increased levels of S100B were found in comparison with controls or patients with pharmacoresistant epilepsy [70]. Indeed, the administration of arundic acid (an S100B inhibitor) to murine models of epilepsy reduced neuroinflammation and improved astrocytic function after status epilepticus [71]. 

The finding of significantly higher peripheral S100B concentrations in patients with ADHD in relation to non-related controls in our study is in line with the hypothesis of global neuroinflammation leading to the dopaminergic disruption observed in this disorder. A pro-inflammatory state in the brain would activate astrocytes, which would react by releasing S100B and activating the NF-kB-dependent inflammatory cascade through the interaction between S100B and RAGE [24,25,26,27]. It should also be considered that S100B secretion occurs not only in astrocytes but also in ependymal cells, endothelial cells, lymphocytes, and several neurons [72]. The positive association between S100B and ADHD symptoms was reported in the study conducted by Liu et al. [44] and corroborated by preclinical studies suggesting the connection between S100B in dopaminergic neurotransmissions, such as the aforementioned by Zhu et al. [30] and the one conducted by Saleh et al. [73]. The latter observed a significant decrease in DA levels, together with an increase in S100B concentrations, in male rats to which the neurotoxic chromium hexavalent was administered. The addition of the neuroprotective sodium alginate led to a recovery in DA concentrations and a consequent neutralization of S100B levels. 

The most striking result was a paradoxically significant increase in S100B concentrations three months after starting therapy (T3) compared to baseline (T0) in the ADHD group, at the same time that a significant improvement in the attention scores (QA/SA) was also observed. In the study conducted by Fernández-López et al. [45], a non-significant increase in S100B levels was reported by our group following MPH treatment, although the lack of noticeable differences was then attributed by the authors themselves to the large dispersion of data. Certainly, we have no clear explanation for the paradoxical increase in S100B levels after three months of treatment onset. The first reason we propose is related to possible changes in DA receptors that modulate the response to MPH: in murine models, D1 and D2 receptors were suggested to play a synergistic role in the sensitization to the therapeutic effects exerted by MPH [74]; thereby, a significant increase in S100B values in the first months after therapy could be linked to MPH-induced overactivation of D2 receptors, given the capacity of S100B to bind this receptor and potentiate its activity [75,76]. A second explanation could be associated with the excess of DA possibly generated by MPH, which has been reported to lead to reactive oxygen species (ROS) production and depletion of the antioxidant glutathione in preclinical studies [77,78,79]. According to this, MPH administration might have originated an initial inflammatory response involving S100B [24,25,26,27] in our patients as a consequence of the increase in DA levels. However, there would not be much congruence between the improvement observed in attentional scores; additionally, the inflammatory reactions following MPH administration have been reported in a few studies on murine models, but there is no definite evidence from human trials. Third, the response of the patients with ADHD and comorbidities to therapy in terms of their S100B concentrations could partly have contributed to the surprising results. In any case, the S100B increase was only slight between T3 and T6, suggesting a plausible attenuating effect exerted by aMT and ω-3 PUFAs on the inflammatory changes following MPH actions, given the aforementioned accretion time in the brain required for the accretion time of ω-3 PUFAs [63]. 

Our study contained several limitations: first, the design did not allow us to observe the relative effect on S100B due to MPH, whereas a clinical trial with different treatment arms would have provided information on the impact of each therapeutic component on this protein; second, we lacked evidence on the response triggered by this treatment in patients with a pure hyperactive/impulsive presentation, as the sample only included patients classified as predominantly inattentive or combined; third, the application of more flexible exclusion criteria with respect to the prior investigations conducted by Oades et al. [42] and Fernández-López et al. [45] may have influenced the S100B levels found in our sample. Certainly, our further investigations in this regard should be accompanied by stricter selection criteria.

To date, this is the first trial reporting on gradual changes in peripheral S100B levels in children and adolescents with ADHD receiving a triple therapy containing MPH and the anti-inflammatory combination of aMT and EPA/DHA. The differences observed between patients with ADHD and controls provide evidence in line with the glial damage leading to dopaminergic disruption and consequent ADHD symptoms [30,73]. No clear explanation was found, however, for the paradoxical increase in S100B values in the first months after treatment, probably attenuated by the anti-inflammatory properties of aMT and EPA/DHA across time. We proposed changes in DA receptors responsible for modulating sensitivity to MPH as one of the reasons, although further studies are required to shed light on the biochemical link between D1/D2 receptors and S100B after administering MPH.

## 5. Conclusions

Our study revealed significantly higher peripheral S100B levels in patients with ADHD compared to controls, strengthening the hypothesis of glial damage within the mechanisms preceding the catecholaminergic disruption responsible for this disorder. Therapeutic administration of MPH, aMT, and ω-3 PUFAs was followed by a paradoxical transient increase in S100B levels, that would require further investigation in future trials with different treatment arms and longer follow-up periods.

## Figures and Tables

**Table 1:** Demographic and clinical information of participants.

	ADHD Group (*n* = 62)	Control Group (*n* = 65)
Age: mean (SD) in years	9.26 (2.11)	9.34 (2.30)
Sex distribution: - Males (%) - Females (%)		
	41 (66.13)	42 (64.61)
	21 (33.87)	23 (35.38)
ADHD presentation: - ADHD-I (%) - ADHD-C (%)		
	32 (51.61)	-
	30 (48.38)	
Comorbidities: - Dyslexia (%) - Primary enuresis (%) - Epilepsy (%) - Migraine (%) - Tics (%)		
	10 (16.13)	-
	4 (6.45)	
	2 (3.22)	
	1 (1.61)	
	1 (1.61)	

**Table 2:** Intergroup and intragroup comparisons of S100B levels for the different ADHD presentations.

	Serum S100B Levels in µg/L: Mean (SD)			Intragroup Comparisons
	T0	T3	T6	
ADHD-I	0.226 (0.093)	0.3117 (0.078)	0.3619 (0.197)	*p* < 0.001 *
ADHD-C	0.2208 (0.087)	0.3617 (0.2023)	0.3177 (0.0866)	*p* < 0.001 *
Intergroup Comparisons	ns	ns	ns	

* Significant results (*p* < 0.001) for comparisons of S100B levels between T0–T3 and T0–T6. No significant differences between T3–T6 for the same ADHD presentation; ns: non-significant.

## Data Availability

Anonymized data related to the current study are available from the corresponding author, upon reasonable request.
